# Teachers’ Growth Mindset, Perceived School Climate, and Perceived Parental Autonomy Support Moderate the Relationship Between Students’ Growth Mindset and Academic Achievement

**DOI:** 10.3390/jintelligence13010008

**Published:** 2025-01-10

**Authors:** Kai Zhang, Wu-Jing He

**Affiliations:** Department of Special Education and Counselling, The Education University of Hong Kong, Hong Kong SAR, China

**Keywords:** growth mindset, academic achievement, teachers’ mindset, school climate, perceived parental autonomy support

## Abstract

This study investigates the moderating effects of several contextual factors (i.e., teachers’ growth mindset, perceived school climate, and perceived parental autonomy support) on the relationship between students’ growth mindset and academic achievement. Drawing on Dweck’s growth mindset theory and recent research findings that highlight the context sensitivity of the growth mindset, we hypothesize that supportive environments strengthen the positive impact of students’ growth mindset on academic outcomes. A sample of 358 middle school students (53.8% female; Mage = 13.38 years, SD = 2.20) from public schools in Shanghai City, mainland China, was assessed via three validated instruments: (1) the Growth Mindset Inventory, which is used to measure students’ and teachers’ beliefs about intelligence; (2) the Delaware School Climate Survey for Students, which is used to assess students’ perceptions of the school climate; and (3) the Perceived Parental Autonomy Support Scale, which is used to evaluate students’ perceived parental autonomy support. Academic achievement was measured by district-level final exam scores. The results of hierarchical regression analyses revealed that teachers’ growth mindset, perceived school climate support (e.g., teacher–student and student–student relations, fairness of rules, school safety, liking of school), and the perception of positive parental autonomy support (e.g., choice, rationale, acknowledgment) positively moderated the relationship between students’ growth mindset and academic achievement. In contrast, the perception of negative parental autonomy factors (e.g., punishment threats, performance pressure, guilt-inducing criticism) negatively moderated this relationship. These results indicate that the relationship between students’ growth mindset and academic achievement may vary depending on contextual factors, highlighting the importance of considering both positive and negative influences when designing educational strategies.

## 1. Introduction

Research on the growth mindset has increasingly shifted toward a deeper exploration of cross-cultural compatibility and the role of contextual factors in influencing mindset development, particularly in light of emerging counterintuitive findings (Burnette et al. 2023). This evolving focus, captured by the mindset-plus-supportive-context framework (Yeager et al. 2022), underscores the importance of embedding mindset interventions within the broader cultural and contextual landscape. However, despite these theoretical advancements, there remains a need for more empirical evidence to enable a full understanding of the contextual heterogeneity of various learning environments, which may influence the successful replication of growth mindset interventions and their predictive power regarding learning outcomes. In response to this need, the present study explores the moderating effects of several contextual factors, including teachers’ mindset (Kroeper et al. 2022), perceived school climate support (Bear et al. 2011), and perceived parental autonomy support (Mageau et al. 2015). These factors have all been highlighted as being closely related to the development of a growth mindset in recent research findings (e.g., Ma et al. 2020; Macnamara and Burgoyne 2023). This study expects that supportive contexts—teachers’ growth mindset, perceived school climate, and perceived parental autonomy support—will moderate the relationship between students’ growth mindset and academic achievement.

### 1.1. Contextual Sensitivity of the Growth Mindset

Dweck’s (2006) mindset theory initially posited that students who believe their intelligence can grow through effort tend to perform better academically. Some studies have indeed shown a positive effect of having a growth mindset on academic achievement (e.g., Polirstok 2017). However, many other studies have presented inconsistent findings, illustrating that the effect of a growth mindset is not always robust or replicable across different populations and contexts; these include large-scale studies such as international comparative research (e.g., Sun et al. 2021; Yeager and Dweck 2020) and meta-analytical studies (e.g., Macnamara and Burgoyne 2023). These inconsistencies emphasize the importance of contextual adaptability in understanding the dynamics of mindset effects, an increasing number of researchers have highlighted the importance of investigating possible contextual heterogeneity in explaining such inconsistent findings regarding the effect of a growth mindset on academic achievement. For example, Yeager et al. (2022) proposed that teachers’ mindsets are a critical factor in determining the results of their students’ growth mindset. Other researchers have also highlighted that perceived school climate support (e.g., Zhang and He 2024) and perceived parental autonomy support (e.g., Chen et al. 2024) are closely related to achieving effective outcomes of a growth mindset. Collectively, these research findings suggest that the capacity to adapt to the needs of the context is more important than endorsing a single mindset independently of the context. This focus on contextual adaptability helps explain the variability in the effectiveness of growth mindset interventions across different settings.

In fact, when Dweck (1999) first proposed mindset theory, certain contextual factors were identified for developing or encouraging the formation of a growth mindset, although she did not discuss them at length. Specifically, Dweck (1999) developed her growth mindset theory in light of Bandura’s (1986) Triadic Reciprocal Determination (TRD) model within the framework of social cognitive theory, which emphasizes the reciprocal dynamics among behavioral, environmental/contextual, and personal factors. In particular, growth mindset theory posits that an individual’s beliefs about intelligence are shaped by the context of the environment, such as feedback from parents and teachers. For example, parents’ praise (context) can influence children’s beliefs about intelligence, which then affects their behavioral outcomes, such as persistence in academic tasks (Gunderson et al. 2013). This theoretical perspective underscores the importance of social and contextual factors in shaping one’s belief in the ability to grow. More critically, it highlights the need for a flexible, context-sensitive approach to leveraging growth mindset theory, particularly in diverse educational settings. Empirical studies have increasingly revealed cultural heterogeneity in terms of the effect of a growth mindset on academic achievement (e.g., Zhang et al. 2020), further underscoring the importance of tailoring growth mindset interventions to the unique demands of the context.

### 1.2. Supportive Contexts for Students’ Growth Mindset

In school settings, recent research has identified several important contextual factors with a significant effect on the formation of students’ growth mindset and the effectiveness of such a mindset. These contextual factors are expected to have a significant moderation effect on the link between students’ mindset and their academic outcomes by amplifying, diminishing, or even changing the direction of the impact of the growth mindset on academic outcomes. This is elaborated below.

#### 1.2.1. Teachers’ Mindsets

Research has increasingly highlighted the pivotal role of teachers’ mindsets in shaping students’ growth mindset, which could significantly influence the way students perceive their own abilities and approach academic challenges (Vestad and Bru 2024). Teachers who believe that abilities can develop through effort tend to provide encouraging feedback and create classroom environments that reinforce these beliefs in students, fostering a growth mindset (Mesler et al. 2021). This process of reinforcing a growth mindset highlights the importance of contextual factors, as the teacher’s mindset serves as a moderator that influences how students interpret and internalize these beliefs. This alignment between teacher and student beliefs creates a positive feedback loop, increasing students’ confidence and resilience, which ultimately contributes to improved academic performance. Yeager et al. (2022) demonstrated that growth mindset interventions are most effective when implemented in environments where teachers themselves hold growth mindsets. In such environments, teachers actively reinforce growth-oriented principles in their teaching practices, providing the support that students need to internalize these beliefs. As a result, students in these settings are more likely to believe in their capacity for growth, leading to better academic outcomes. The interaction between the teacher and student mindsets is thus crucial for fostering a growth-oriented learning environment, where students feel safe in taking risks and embracing challenges.

A practical illustration of this dynamic is provided by Ronkainen et al. (2019), who conducted a case study in a Finnish elementary school. They found that a teacher’s growth mindset was evident in the feedback provided to students, e.g., using phrases such as “not yet” to encourage students to see challenges as growth opportunities. This strategy helped students gradually transition from a fixed mindset to a growth mindset, underscoring the direct impact of teacher behavior on student mindset development and academic performance. Furthermore, Yu et al. (2022) reported that students were more likely to develop a growth mindset in classrooms where teachers used growth-mindset-oriented tasks such as inquiry-based learning techniques that promote exploration, resilience, and an open mind to encourage positive development through effort. Conversely, when teachers used fixed-mindset-oriented tasks such as ability-based learning techniques, students tended to adopt a fixed mindset. These findings illustrate how different instructional strategies can either support or hinder the development of a growth mindset. In another study, Kroeper et al. (2022) identified additional teacher behaviors that signal growth-mindset beliefs to students. Teachers who provided frequent encouraging feedback, responded supportively to struggling students, and prioritized learning over performance were more likely to foster growth mindsets in their students. These behaviors revealed how teachers, through their day-to-day interactions, actively nurtured the development of growth mindsets. Taken together, these studies demonstrate that teachers’ growth-mindset beliefs are critical moderators of the relationship between students’ growth mindset and their academic achievement. Teachers who emphasize effort, focus on growth, and provide supportive and constructive feedback significantly increase the effectiveness of growth mindset interventions, which can lead to better academic outcomes. In this regard, a positive moderation effect of teachers’ growth mindset on the link between students’ growth mindset and academic outcomes is expected.

#### 1.2.2. Perceived School Climate Support

The perceived school climate refers to individuals’ perception of the quality and characteristics of their school environment, including teacher–student relationships, support systems, and institutional structures (Worrell and Hale 2001). This variable was chosen due to its critical role as a contextual moderator that shapes how students interpret and respond to growth mindset interventions. Previous research has consistently demonstrated that a positive school climate provides the psychological safety and encouragement necessary for students to embrace challenges, persevere through setbacks, and develop a growth mindset (Bear et al. 2015; Paunesku et al. 2015). These perceptions shape students’ beliefs about their abilities and their capacity to grow (Yu et al. 2022). Students who perceive their school climate as supportive and inclusive are more likely to embrace challenges and persist through setbacks, which are both key components of a growth mindset. The literature has identified several dimensions of perceived school climate support in school settings, including (1) teacher–student relations, (2) student–student relations, (3) fairness of rules, (4) school safety, and (5) liking of school (Bear et al. 2011).

First, the dimension of teacher–student relations is crucial. Students who experience positive, supportive interactions with teachers tend to feel encouraged to take academic risks and learn from mistakes. In contrast, students who experience distant or disengaged relationships with their teachers may adopt a fixed mindset, seeing challenges as insurmountable barriers (Charlton et al. 2021). Second, the dimension of student–student relations plays a significant role in shaping students’ mindsets. In schools where students perceive a respectful and collaborative peer culture, they are more likely to engage in cooperative learning, reinforcing the belief that intelligence can develop through effort and shared learning experiences (Le et al. 2018). This aligns with the idea that abilities are not fixed but can improve with perseverance (Zeng et al. 2019). Conversely, negative peer interactions—in which competition dominates and success is seen as scarce—may lead to a fixed mindset. In such environments, students may feel threatened by others’ success and avoid academic risk, reinforcing isolation and disengagement (Dweck 2006). Third, with respect to the fairness of rules, when students perceive school rules and disciplinary actions as fair and consistently applied, they are more likely to engage positively with their learning environment (Özer and Demirtaş 2010). Fairness fosters a sense of trust in the system and encourages students to persist in the face of challenges, knowing that their efforts will be justly recognized. This perception is in line with the growth mindset, wherein effort leads to improvement and setbacks are part of the learning process (Mrazek et al. 2018). In contrast, perceptions of unfairness may undermine students’ motivation and lead to disengagement, contributing to the adoption of a fixed mindset (Yeager and Dweck 2012).

Fourth, school safety is integral to creating an environment in which students feel secure enough to take risks, make mistakes, and embrace learning challenges. When students perceive their schools as safe, they are more likely to step out of their comfort zones, viewing challenges as opportunities for growth rather than threats to their self-worth (Timm 2015). Conversely, schools that are perceived as unsafe or chaotic may encourage a defensive, risk-averse attitude, which can stifle the development of a growth mindset (Hughes et al. 2015). In such environments, students might avoid challenges altogether, fearing failure or negative consequences. Finally, the fifth dimension—liking of school—reflects overall student satisfaction with their school experience. Students who enjoy school and feel proud of their environment tend to be more motivated and resilient when facing academic challenges (Willingham 2021). A positive connection to the school community can also foster a growth mindset, as students are more likely to embrace challenges and view them as opportunities for personal development when they feel positively toward their learning environment. Additionally, students who feel involved in shaping school decisions—such as by joining student councils or providing feedback—are more likely to perceive their school as a place where their voice matters (Lowery 2008). This sense of agency aligns with growth mindset principles, which emphasize owning one’s learning and viewing challenges as opportunities for personal growth (Mager and Nowak 2012). In contrast, dissatisfaction with school can weaken students’ connection to their learning environment and foster a fixed mindset. Students who feel disengaged or disconnected from their school community may be less inclined to view academic challenges as meaningful or worth their effort (Fredricks et al. 2004). Instead of seeing setbacks as opportunities for growth, they may view them as reflections of their own inherent limitations, leading to decreased motivation and persistence. Such disengagement can further reinforce alienation from school, making students less likely to participate in decision-making processes and thereby deepening their disconnection.

In summary, these findings generally suggest a positive moderating role of perceived school support across five dimensions (i.e., teacher–student relations, student–student relations, fairness of rules, school safety, and liking of school) with respect to the effect of students’ growth mindset on their academic outcomes.

#### 1.2.3. Perceived Parental Autonomy Support

Perceived parental autonomy support refers to the extent to which students perceive their parents as encouraging independence, providing choices, and fostering self-regulation in their learning (Fousiani et al. 2014). This variable was selected because it serves as a key contextual moderator that influences how effectively students internalize and apply growth mindset principles. Autonomy-supportive parenting has been shown to promote intrinsic motivation, resilience, and adaptive learning strategies (Fousiani et al. 2014; Won and Shirley 2018), all of which are closely linked to the development of a growth mindset. This support involves behaviors such as offering choices, acknowledging students’ psychological needs, and promoting self-regulation. Recent research by Ma et al. (2020) indicates that perceived parental autonomy support positively influences students’ growth mindset. By fostering an environment in which students feel empowered to take ownership of their learning, autonomy-supportive parents encourage students to make independent decisions and embrace challenges, thereby promoting persistence through difficulties and viewing failure as an opportunity for growth. The key factors identified in the literature (Mageau et al. 2015) that contribute to students’ perceptions of autonomy support include (1) choice within certain limits; (2) rationale for demands and limits; (3) acknowledgment of feelings; and avoidance of controlling behaviors such as (4) threats of punishment, (5) performance pressure, and (6) guilt-inducing criticism.

For example, providing students with choices within set boundaries promotes a sense of autonomy while maintaining a necessary structure, which has been shown to enhance students’ ownership of their learning and foster a growth mindset (Reeve 2021). Posthumus and Kleinhans (2014) further emphasize that even in environments with external constraints, offering limited choices enables students to exercise agency and approach challenges with a mindset focused on growth. Providing a clear rationale for rules and demands also plays a crucial role. Jang et al. (2010) report that when students are given explanations for why certain limits are necessary, they are more likely to internalize these demands, leading to greater engagement and motivation. Similarly, Reeve (2021) argue that rationales help students perceive rules as supportive rather than controlling, fostering an environment conducive to resilience and growth. This aligns with findings by Johansen et al. (2023), who demonstrate that students in autonomy-supportive environments—where their feelings are acknowledged and rationales are provided—tend to develop higher levels of intrinsic motivation and academic performance. Acknowledgment of feelings is another critical component of parental autonomy support. Froiland and Worrell (2017) highlight that when parents validate their children’s emotions, they foster a psychologically safe environment, allowing students to express frustrations and approach challenges as growth opportunities. Such emotional support is linked to higher intrinsic motivation and academic aspirations, which are key elements of a growth mindset. Such emotional support is linked to higher intrinsic motivation and academic aspirations, which are key elements of a growth mindset. Moè and Katz (2018) further emphasize the role of parental emotions in shaping students’ emotional responses through self-efficacy. Their findings suggest that parents’ positive emotions and autonomous motivation are associated with higher levels of students’ self-efficacy, which in turn supports adaptive emotional and motivational outcomes during academic tasks. This underscores the importance of parents creating an emotionally supportive and autonomy-promoting atmosphere to enhance their children’s academic engagement and resilience.

However, when parents rely on threats of punishment to control behavior, as Katz et al. (2018) note, students may associate failure with negative consequences, leading them to adopt a fixed mindset and avoid risks. Moreover, perceived performance pressure from parents also plays a significant role in shaping whether students develop a growth mindset or a fixed mindset. Pedersen (2017) suggests that when parents prioritize academic outcomes such as grades over the learning process, students may feel that their worth is tied solely to their performance. This emphasis on results can foster a fixed mindset, in which students perceive their abilities as limited. In contrast, reducing performance pressure may help students feel safe in taking risks and learning from mistakes, which is associated with the development of a growth mindset. Finally, guilt-inducing criticism, in which parents use guilt to influence behavior, can further undermine students’ sense of autonomy and resilience. Assor et al. (2004) report that controlling parental behaviors, including guilt-inducing criticism, reduce students’ intrinsic motivation and their willingness to engage with challenges. Brooks (2014) similarly reports that guilt-inducing criticism often leads to anxiety, which hinders students’ persistence and ability to view challenges positively. Mageau et al. (2015) support this, showing that controlling strategies, including guilt-inducing tactics, decrease students’ motivation and self-regulation, reinforcing a fixed mindset.

In summary, the research findings suggest a domain-specific pattern with respect to the moderating role of perceived parental autonomy support in determining the effect of students’ growth mindset on their academic outcomes. Specifically, three dimensions of autonomy support (i.e., perceived choice within certain limits, rationale for demands and limits, and acknowledgment of feelings) are expected to have a positive moderating role that may increase the strength of the relationship between the growth mindset and academic achievement; however, the other three dimensions of autonomy support (i.e., perceived threats of punishment, performance pressure, and guilt-inducing criticism) are expected to play a negative moderating role that may weaken this relationship.

### 1.3. The Present Study

Contextual heterogeneity has been suggested as an explanation for the inconsistent findings regarding growth mindset interventions. Research shows that contextual factors can strengthen or weaken their impact on academic outcomes (Macnamara and Burgoyne 2023; Yeager et al. 2022). The present study extends this understanding by exploring three potential moderators—teacher mindset, perceived school climate, and perceived parental autonomy support—in the relationship between students’ growth mindset and academic achievement. This study aims to provide a more nuanced understanding of how supportive environments influence this relationship. Derived from the perspective of the mindset-plus-supportive-context framework and relevant research, the following study hypotheses are formulated:

Hypothesis 1 (H1) posits that teachers’ growth mindset positively moderates the relationship between students’ growth mindset and academic achievement.

Hypothesis 2 (H2) posits that students’ perceived school climate support positively moderates the relationship between students’ growth mindset and academic achievement.

Hypothesis 3 (H3) posits a dimension-specific moderating effect of perceived parental autonomy support on the relationship between students’ growth mindset and academic achievement. Specifically, H3a–c postulate that certain dimensions of parental autonomy support—perceived choice within limits, rationale for demands, and acknowledgment of feelings—positively moderate the relationship between students’ growth mindset and academic achievement. Conversely, H3d–f posit that dimensions such as perceived threats of punishment, performance pressure, and guilt-inducing criticism negatively moderate this relationship.

## 2. Methods

### 2.1. Participants and Procedures

An initial sample of 373 middle school students and 63 teachers was recruited from public middle schools in various districts of Shanghai. Of these, 15 students were excluded from the data analysis because of incomplete responses (attrition rate = 4.0%), which is considered acceptable, as Rubin and Babbie (2016) suggest that attrition rates under 20% are typically acceptable in social science research. The final student sample consisted of 358 participants (53.8% female; Mage = 13.38 years, SD = 2.20, range = 12–15 years; educational years = 7.80 years, SD = 1.70, range = 7 to 9 years). The teacher sample included 63 participants (57.8% female; Mage = 35.42 years, SD = 6.80, range = 28–52 years), consisting primarily of homeroom teachers and subject teachers who directly interacted with the participating students. All participants were ethnically Chinese and primarily came from middle- to lower-middle-class socioeconomic backgrounds. Participation was entirely voluntary, this classification was based on the socioeconomic characteristics of the school districts where participants were recruited, which are predominantly attended by families within these income brackets. Prior to data collection, the participating schools, teachers, students, and their parents were fully informed about the study’s objectives, procedures, confidentiality, and anonymity guarantees. Students, their parents, teachers, and schools provided informed consent to participate in the study. Data collection was conducted in group settings, with approximately 30–40 student participants per session, either in classrooms or conference rooms at the schools. The assessments for students included the Growth Mindset Inventory (GMI), the Perceived School Climate Scale (PSCS), and the Parental Autonomy Support Questionnaire (PASQ). Teachers completed the Growth Mindset Inventory (GMI) to assess their beliefs about intelligence and learning. Each questionnaire took approximately 5–10 min to complete, with the total session lasting around 25–30 min. Standardized instructions were provided to ensure consistency across sessions, with research assistants onsite to facilitate the process. The entire procedure was designed to ensure a calm and focused environment conducive to participant engagement. Additionally, Students’ academic achievement was assessed using district-level final exam scores on a 100-point scale. These scores were sourced directly from official transcripts provided by the participating schools, ensuring both the accuracy and completeness of the data. Teachers’ growth mindset data were collected using the GMI, and their responses were linked to the students they taught to explore the moderating effects of teachers’ growth mindset on the relationship between students’ growth mindset and academic performance. The research team collected all data after written consent was obtained from both the schools and the participants.

### 2.2. Instruments

#### 2.2.1. Growth Mindset Scale

Both participating students’ and teachers’ growth mindsets were measured via the Chinese-adapted version of the growth mindset subscale, the Growth Mindset Inventory (GMI; Dweck 1999), which assesses individuals’ mindsets about the malleability of intelligence. This 4-item inventory uses a Likert scale ranging from 1 (strongly disagree) to 6 (strongly agree) and includes statements such as “Even your basic intelligence level can be increased considerably.” A higher score indicates a greater degree of endorsement of the idea that intelligence is malleable. The GMI effectively mitigates the positive wording effect highlighted in recent literature (Yu and Kreijkes 2017), which can lead individuals to identify as incremental theorists due to social desirability bias, especially those familiar with the growth mindset concept included in the questionnaire (Song 2018). The GMI has been shown to be reliable in both Chinese and international contexts, with values of Cronbach’s α ranging from 0.76 to 0.83 (Wang et al. 2021; Zhang and He 2024). In this study, the GMI demonstrated similarly acceptable reliability, with Cronbach’s α = 0.82.

#### 2.2.2. Perceived School Climate Support Scale

The Chinese-adapted version of the Delaware School Climate Survey for Students (DSC-S; Bear et al. 2011) was employed in this study to assess participants’ perceptions of school climate support. The DSC-S consists of 23 items designed to capture five core dimensions of school climate: (1) teacher–student relations (e.g., teachers care about their students), (2) student–student relations (e.g., students treat each other with respect), (3) fairness of rules (e.g., the school’s Code of Conduct is fair), (4) school safety (e.g., I feel safe in this school), and (5) liking of the school (e.g., I am proud of my school). The participants responded to these items on a four-point Likert scale ranging from “strongly disagree” (1) to “strongly agree” (4), which enabled a detailed analysis of their views on various aspects of the school climate. In a recent study with Serbian high school students, Đorđić et al. (2022) reported a Cronbach’s alpha of 0.74 for the DSCS-S, indicating acceptable internal consistency. Similarly, in the present study, the DSCS-S demonstrated acceptable reliability, with a Cronbach’s α of 0.85.

#### 2.2.3. Perceived Parental Autonomy Support Scale

The Perceived Parental Autonomy Support Scale (P-PASS; Mageau et al. 2015) was used to assess participants’ perceptions of parental autonomy support. The scale consists of 24 items divided into six subdimensions: (1) choice within certain limits is measured by items such as “My parents encouraged me to make choices in line with my values”; (2) rationale for demands and limits is captured by statements such as “My parents explained the reasons behind their decisions and limits”; (3) for acknowledgment of feelings, an example item is “My parents were able to put themselves in my shoes and understand my feelings”; (4) threats of punishment are assessed with items such as “As soon as I didn’t do exactly what my parents wanted, they threatened to punish me”; (5) performance pressures are reflected by items such as “My parents insisted that I always be better than others”; and (6) guilt-inducing criticism is measured with statements such as “When my parents wanted me to act differently, they made me feel ashamed in order to make me change.” Participants rated each item on a 7-point Likert scale ranging from “Do not agree at all” (1) to “Very strongly agree” (7). A recent study by Inda Caro et al. (2022) supported the reliability of the P-PASS, obtaining Cronbach’s alpha values between 0.89 and 0.94. In the present study, the P-PASS demonstrated acceptable reliability, with a Cronbach’s alpha of 0.88.

#### 2.2.4. Data Analysis

SPSS software (version 26.0) was used for the statistical analysis in this study. Before hypothesis testing, a Pearson correlation analysis was conducted to identify the bivariate correlations among the main study variables (i.e., students’ growth mindset, teachers’ growth mindset, perceived school climate support, perceived parental autonomy support, and academic achievement). The interpretation of the correlation coefficients followed Cohen’s (1988) guidelines, which define *r* ≥ 0.10 (or ≤−0.10) as indicating a small effect, *r* ≥ 0.30 (or ≤−0.30) as indicating a medium effect, and *r* ≥ 0.50 (or ≤−0.50) as indicating a large effect. In addition to the main variables, control variables such as age and sex were included to assess their potential influence on the results, ensuring a thorough examination of the relationships among the study variables.

To test the hypotheses with respect to the moderating effects of teacher mindset (H1), perceived school climate support (H2), and perceived parental autonomy support on the growth mindset−academic achievement relationship (H3) among students, hierarchical regression analyses were conducted. To test each of these three hypotheses, three steps were involved in the analyses. In Step 1 (Model 1), demographic variables such as age, gender, and education level were included to control for their potential influence on academic achievement. In Step 2 (Model 2), the direct effect of students’ growth mindset on academic achievement was examined. In Step 3 (Model 3), interaction terms between students’ growth mindset and each of the three contextual factors (i.e., teachers’ growth mindset, perceived school climate support, and perceived parental autonomy support) were added to assess their moderating effects on the relationship between growth mindset and academic achievement. Prior to running the regression, key assumptions were evaluated to ensure the reliability of the analyses. The normality of the residuals was assessed via skewness and kurtosis values, both of which were within acceptable ranges (skewness: −0.24 to 0.89; kurtosis: −0.31 to 1.10), confirming that there were no serious deviations from normality. Homoscedasticity was tested via the Breusch–Pagan test, which yielded nonsignificant results (*p* = 0.36), confirming that the variance of the residuals was consistent across the levels of the independent variables. Multicollinearity was assessed via variance inflation factor (VIF) statistics; all VIF values were below 2.0, ranging from 1.05 to 1.32, indicating no multicollinearity issues (Johnston et al. 2018). In addition to the regression analyses, simple slope plots were generated to visually represent the moderation effects, illustrating how academic achievement varied under high (*M* + 1*SD*) and low (*M* − 1*SD*) levels of the moderating variables. These plots followed standard practices in similar research, providing a clear visual representation of the moderation effects. All analyses were conducted with a significance threshold of *p* < 0.05.

## 3. Results

### 3.1. Descriptive Statistics and Bivariate Correlations

Table 1, Table 2 and Table 3 present the descriptive statistics and bivariate correlations of the study variables. The mean score for student mindset was 5.21 (*SD* = 1.57), and the mean score for teacher mindset was 5.15 (*SD* = 1.32). Academic achievement had a mean score of 82.18 (*SD* = 9.18). The mean scores for the five dimensions of perceived school climate support ranged between 2.58 (for school safety; *SD* = 0.81) and 3.19 (for student–student relations; *SD* = 0.75). The mean scores for the six dimensions of perceived parental autonomy support ranged between 4.31 (for rationale for demands and limits; *SD* = 0.61) and 6.53 (for performance pressures; *SD* = 1.21). With respect to bivariate correlations, students’ growth mindset was positively and significantly correlated with academic achievement (*r* = 0.58, *p* < 0.001). Regarding H1, students’ growth mindset was positively correlated with teachers’ growth mindset (*r* = 0.48, *p* < 0.005). Regarding H2, students’ growth mindset was positively correlated with all five dimensions of perceived school climate support (*r* = 0.15–0.35, all *p* values < 0.01). Regarding H3a–3f, students’ growth mindset was found to be positively and significantly correlated with three positive dimensions of perceived parental autonomy support: choice within certain limits (*r* = 0.29, *p* < 0.05), rationale for demands and limits (*r* = 0.31, *p* < 0.01), and acknowledgment of feelings (*r* = 0.26, *p* < 0.05); however, it was negatively and significantly correlated with three negative dimensions of perceived parental autonomy support: threats of punishment (*r* = −0.41, *p* < 0.01), performance pressure (*r* = −0.45, *p* < 0.01), and guilt-inducing criticism (*r* = −0.42, *p* < 0.01).

### 3.2. Regression Analyses

**Figure 1:**
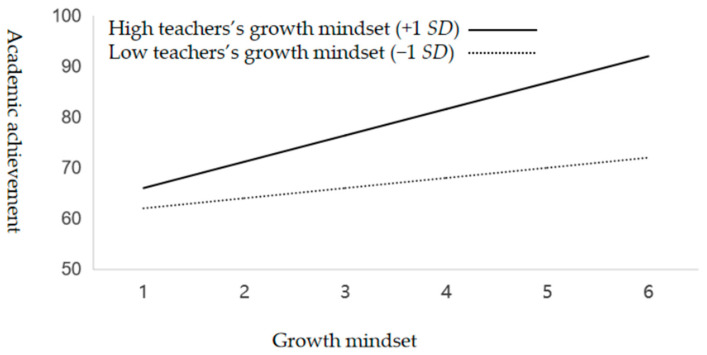
Teachers’ growth mindset moderated the relationship between students’ growth mindset and academic achievement.

Table 4, Table 5 and Table 6 present the results of regression analyses with respect to testing the moderation effects of teachers’ growth mindset (H1), perceived school climate support (H2), and perceived parental autonomy support (H3a–3f) on the relationship between students’ growth mindset and academic achievement. As shown in Table 2, students’ growth mindset was positively predictive of academic achievement in Model 2/M2 (Δ*R*^2^ = 0.12, Δ*F* = 27.37, *p* < 0.001) after controlling for the possible covariate effect of the demographic variables in Model 1/M1 (*R*^2^ = 0.02, *F* = 3.91, *p* = 0.13). Moreover, the results of Model 3/M3 revealed a significant increase in explanatory power (Δ*R*^2^ = 0.09, Δ*F* = 12.51, *p* < 0.001) with the introduction of the interaction term for students’ growth mindset × teachers’ growth mindset, confirming H1 with respect to the positive moderation effect of teachers’ growth mindset on the relationship between students’ growth mindset and their academic achievement. Figure 1 visually illustrates this moderation effect, showing a relatively steep slope of the relationship between student growth mindset and academic performance for the students who reported high teacher growth mindset; in contrast, this relationship has a relatively flat slope for those reporting low teacher growth mindset. These results confirm H1 by revealing that a higher level of teacher growth mindset could strengthen the positive effect of students’ growth mindset on their academic performance.

**Figure 2:**
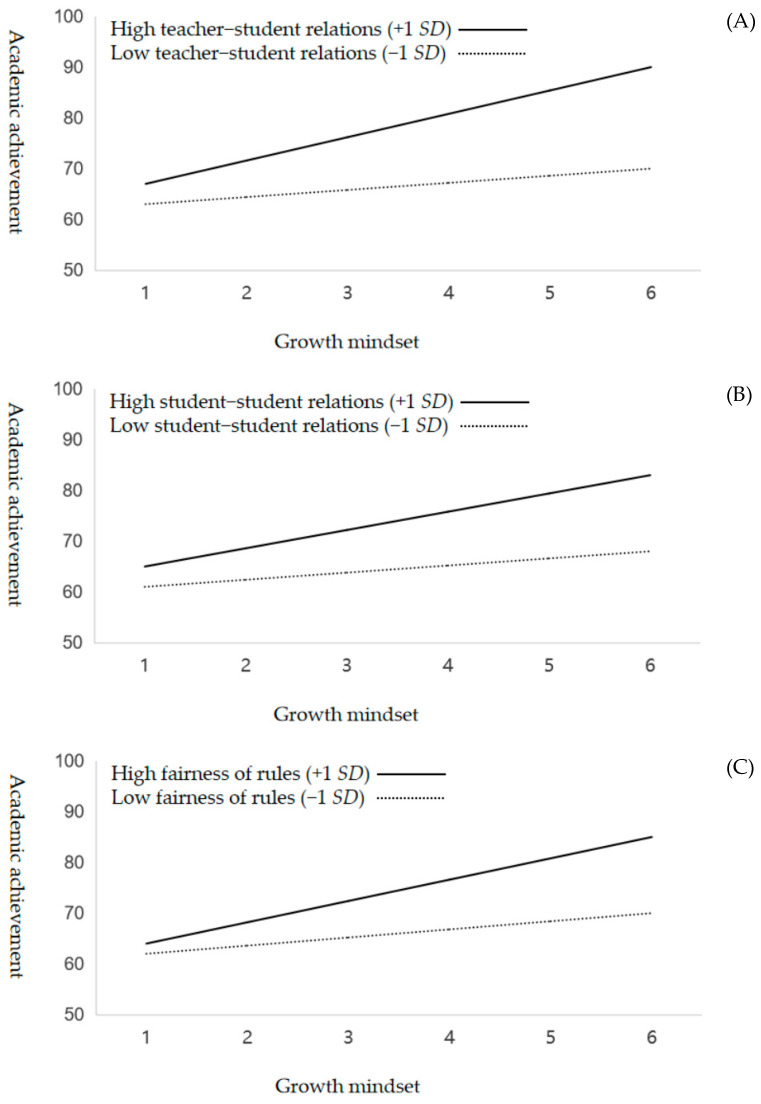

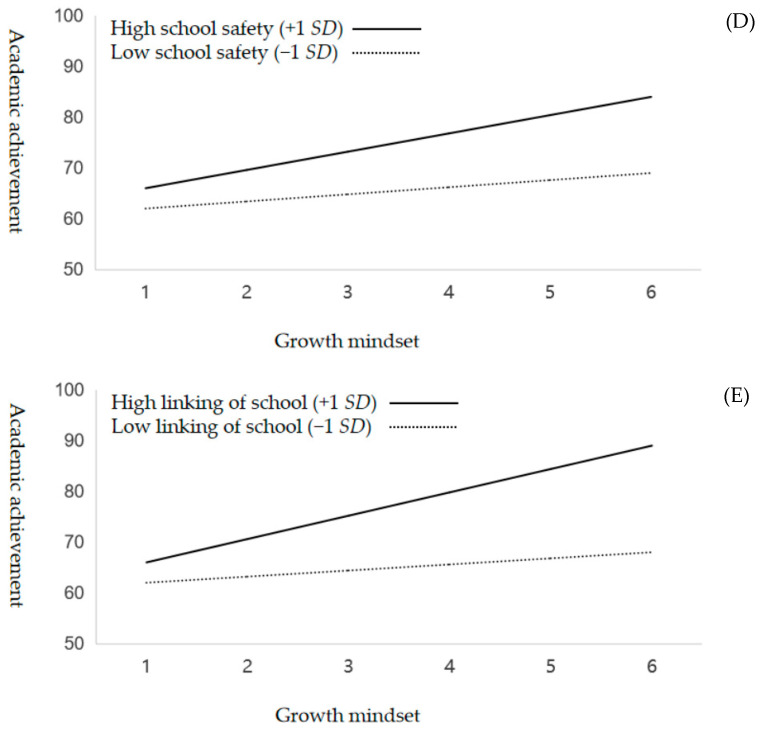
Five dimensions of perceived school climate—teacher–student relations (Panel **A**), student–student relations (Panel **B**), fairness of rules (Panel **C**), school safety (Panel **D**), and liking of school (Panel **E**)—positively moderated the relationship between a growth mindset and academic achievement.

With respect to H2, regarding the positive moderation effect of perceived school climate support, the results of Model 3/M3 shown in Table 3 also revealed a significant increase in the explanatory power of the model (Δ*R*^2^ = 0.10, Δ*F* = 16.05, *p* < 0.001) upon introducing the interaction terms of students’ growth mindset and all five dimensions of perceived school climate support (β = 0.11–0.16, all *p* values < 0.01). These results confirmed H2. The results of all five panels in Figure 2 visually depict a general trend of a relatively steep slope for the students who reported high perceived school climate support and a relatively flat slope for those who reported low perceived school climate support. These results are in accordance with H2, that the perception of school climate support (in terms of teacher–student relations, student–student relations, fairness of rules, school safety, and liking of school) can strengthen the positive effect of students’ growth mindset on their academic performance.

**Figure 3:**
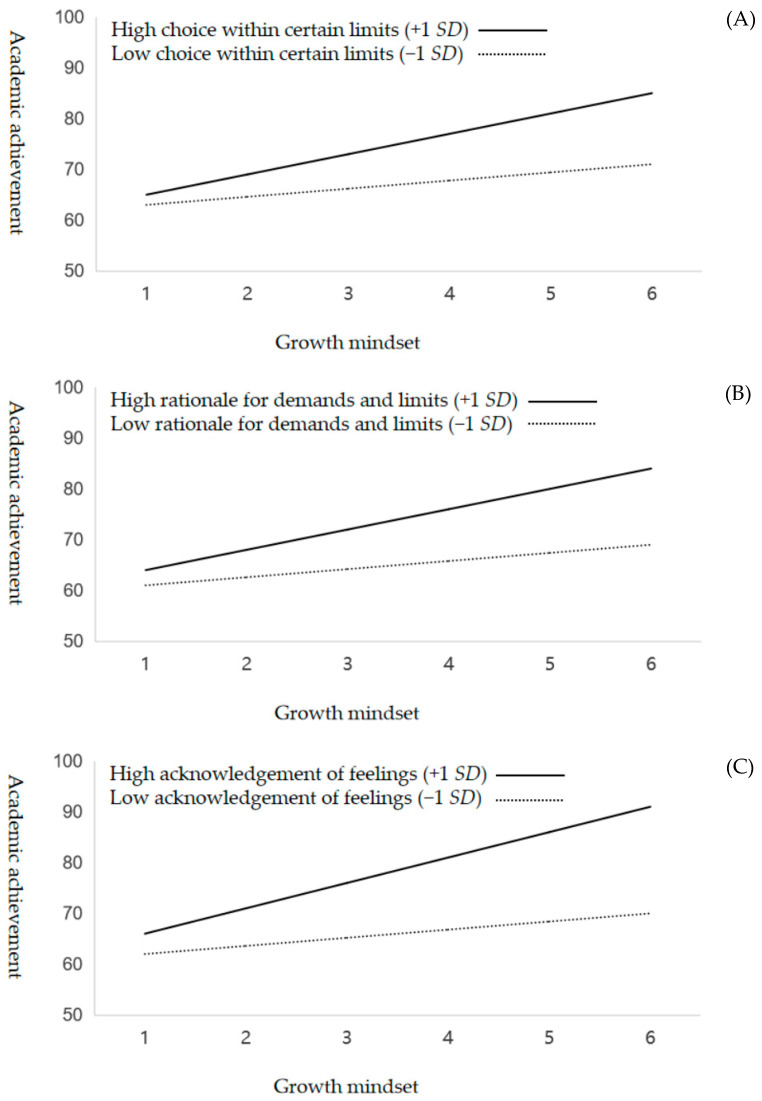

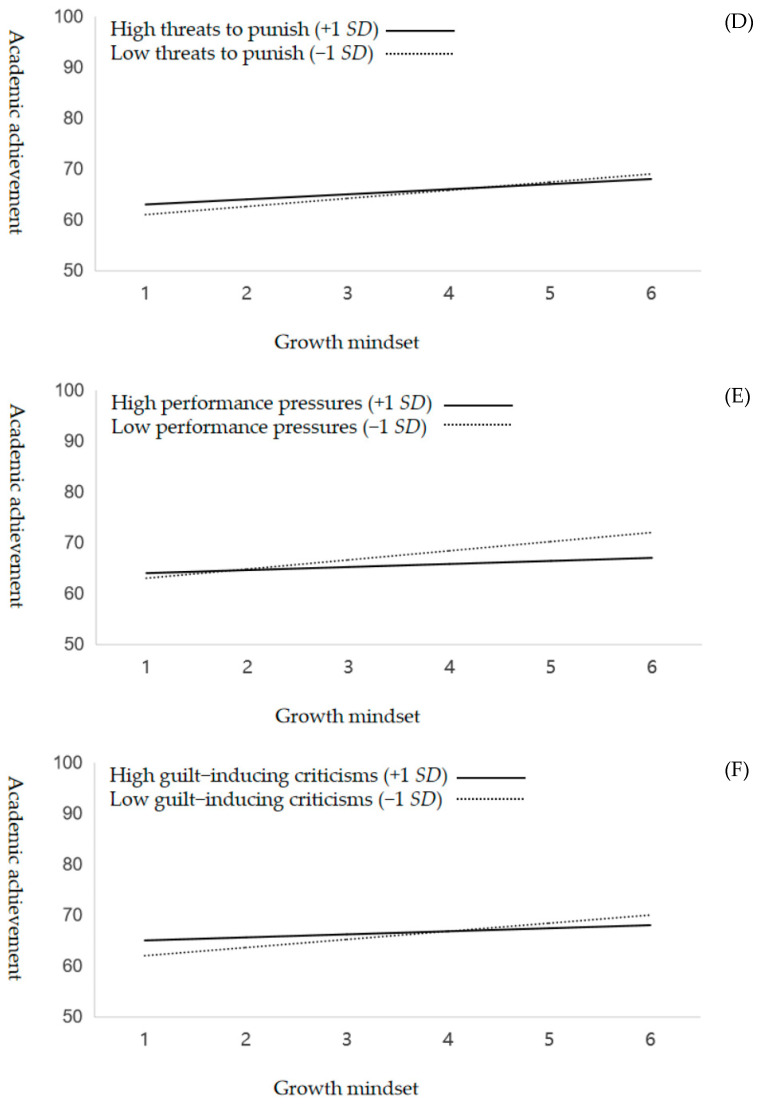
Three dimensions of perceived parental autonomy support—choice within certain limits (Panel **A**), rationale for demands and limits (Panel **B**), and acknowledgment of feelings (Panel **C**)—positively moderated the relationship between a growth mindset and academic achievement. The other three dimensions—threats of punishment (Panel **D**), performance pressure (Panel **E**), and guilt-inducing criticism (Panel **F**)—negatively moderated the relationship between a growth mindset and academic achievement.

With respect to H3, regarding the domain-specific pattern of the moderating effect of perceived parental autonomy support, the results of Model 3/M3 in Table 4 show that the three positive dimensions of perceived parental autonomy support (i.e., choice within certain limits, rationale for demands and limits, acknowledgment of feelings) had a positive moderation effect on the relationship between students’ growth mindset and their academic performance (β = 0.12–0.16, all *p* values < 0.01). In contrast, the three negative dimensions of perceived parental autonomy support (i.e., threats of punishment, performance pressure, guilt-inducing criticism) had a negative moderation effect on the relationship (β = −0.11–−0.12, all *p* values < 0.01). Overall, the results of the model revealed a significant increase in the explanatory power of the model (Δ*R*^2^ = 0.13, Δ*F* = 32.61, *p* < 0.001) upon introducing the interaction terms of students’ growth mindset and all six dimensions of perceived parental autonomy support. Moreover, Figure 3 indicates an amplifying effect of perceived parental autonomy support in terms of choice within certain limits, rationale for demands and limits, and acknowledgment of feelings with respect to the relationship between students’ growth mindset and academic performance. However, it depicts a diminishing effect on this relationship of perceived parental autonomy support in terms of threats of punishment, performance pressure, and guilt-inducing criticism.

## 4. Discussion

### 4.1. Theoretical Implications

This study deepens our understanding of how contextual factors—particularly teacher mindset, perceived school climate, and parental autonomy support—moderate the relationship between students’ growth mindset and their academic achievement. Recent work on mindset theory increasingly emphasizes the importance of environmental and contextual factors in shaping how growth mindsets are developed and implemented in educational settings (Burnette et al. 2023; Kroeper et al. 2022). While the findings align with the mindset-plus-supportive-context framework (Yeager et al. 2022), the evidence from this study is correlational and thus does not establish causal relationships. This limitation underscores the need for caution when interpreting how contextual factors amplify or diminish the effects of a growth mindset. Teachers’ growth-mindset beliefs are associated with students’ mindset formation and application, as teachers may create classroom environments that nurture persistence and learning through effort (Mesler et al. 2021). When teachers themselves demonstrate a growth mindset, their beliefs are linked to fostering an environment that supports the internalization of these beliefs and are associated with improved academic performance. However, while these findings reinforce prior research, the relatively high average scores for teachers’ growth mindset in this study suggest a potential ceiling effect that may limit the variability needed to fully understand the range of moderating impacts. This result reinforces the findings by Yeager et al. (2022), which show that the effectiveness of growth mindset interventions significantly increases when teachers actively model and promote growth-mindset beliefs. Furthermore, the perceived school climate—particularly dimensions such as teacher–student relations and fairness of rules—was found to strengthen the link between students’ growth mindset and academic achievement. This supports the findings by Bear et al. (2011), who argue that positive school environments, which foster safety, respect, and fairness, provide the necessary psychological foundation for students to embrace academic challenges and persevere. Such environments may provide the psychological safety needed for students to take intellectual risks, a core element of the growth mindset framework. Additionally, positive teacher–student relationships have been shown to promote resilience and engagement, supporting students as they encounter academic difficulties (Zhang and He 2024). This highlights the importance of fostering inclusive school climates, where the principles of effort and growth are actively reinforced. The study also elucidates the crucial role of parental autonomy support in moderating growth mindset outcomes. In addition to exploring the positive relation between perceived parental autonomy support and students’ growth mindset, as found in previous research by Ma et al. (2020). The findings suggest that parents who provide autonomy, such as by offering rationales for decisions and acknowledging emotions, positively moderate their children’s growth mindset and academic achievement. In contrast, controlling parental behaviors, such as performance pressure and guilt-inducing criticism, were negatively associated with students’ growth mindsets, undermining the positive effects of mindset interventions. Collectively, these findings underscore the complex interplay between individual beliefs and environmental support systems in shaping academic outcomes. Growth mindset interventions are most effective when they are integrated with supportive teaching practices, a positive school climate, and autonomy-supportive parental behaviors. By examining the growth mindset within specific contextual factors such as teacher mindset, perceived school climate, and parental autonomy support, this study provides insights into how interventions may be influenced by the environmental contexts of students. Moving forward, future research should explore how specific aspects of school and family environments interact with mindset development to optimize educational outcomes, particularly across different cultural settings.

### 4.2. Practical Implications

The findings of this study underscore the potential importance of creating supportive contexts for fostering students’ growth mindset and enhancing academic achievement. Teachers’ mindsets, the school climate, and parental autonomy support serve as potential moderators in this process. For example, teachers’ mindsets significantly shape how students approach challenges. When teachers adopt a growth mindset, they are more likely to use effective feedback strategies and promote resilience in their students (Mesler et al. 2021). Training programs for teachers can focus on enhancing these beliefs to create growth-oriented classrooms. Research has shown that professional development that encourages a growth mindset in teachers is associated with better student outcomes (Yeager et al. 2022). Schools should thus focus on such teacher training initiatives to amplify the impact of mindset interventions (Yeager et al. 2022). For instance, Burnette et al. (2023) highlight the importance of implementation fidelity in mindset interventions, suggesting that teacher training programs should include clear guidelines and structured activities to ensure consistent application of growth mindset principles. For teachers’ training, incorporating growth mindset principles into pre-service teacher education may ensure these strategies are integrated early in a teacher’s career, creating a foundation for long-term positive impacts on students. Additionally, aligning mindset-focused training with the formal curriculum objectives may be important. Teachers should consider how their mindset beliefs may align with and relate to broader educational goals, such as fostering creativity, critical thinking, or academic excellence. In settings where the curriculum emphasizes competitive outcomes, it might be beneficial for teachers to explore strategies that integrate growth mindset principles while addressing performance-driven objectives. For example, interventions that carefully balance performance goals with personal development could help ensure that curriculum demands are met while promoting adaptive mindset practices. However, it is also important to recognize that endorsing a single mindset universally, without considering the demands of specific educational contexts, may not always yield optimal results. In competitive or high-stakes environments, a fixed mindset may help students focus on performance outcomes, which could align better with the immediate demands of such systems. Therefore, fostering the capacity to adapt mindsets based on contextual needs—rather than strictly promoting one mindset—may ultimately be more beneficial. This adaptability could be an important consideration in the design of future interventions. In addition, a positive school climate fosters an environment in which students feel supported and are more likely to embrace growth-oriented behaviors (Bear et al. 2011). Key dimensions such as teacher–student relations, fairness of rules, and school safety have been identified as influential factors in this process (Bear et al. 2011). Schools can implement programs that strengthen these dimensions, creating an inclusive and supportive learning environment in which students are encouraged to view challenges as growth opportunities (Charlton et al. 2021). This aligns with research by Yu et al. (2022), who reported that supportive school environments enable the development of a growth mindset, particularly in contexts that promote fairness and respect. Moreover, the role of parental autonomy support in enhancing the growth mindset cannot be overlooked. Schools can engage parents through workshops or informational sessions on autonomy-supportive behaviors, helping them understand how minimizing performance pressure and guilt-inducing criticism positively influences their children’s academic motivation (Assor et al. 2004). For instance, parent-focused programs could include interactive role-playing activities to practice autonomy-supportive communication techniques, making the application of these strategies more tangible and effective. Ma et al. (2020) reported that students who perceive higher levels of parental autonomy support demonstrate greater persistence and academic resilience, providing clear implications for fostering a growth-oriented home environment. By aligning these supportive factors—teacher mindset, the school climate, and parental autonomy support—educational institutions may create environments that support students’ growth mindset and contribute to their academic development. Future research should also explore how different mindsets can be flexibly applied in various educational systems to balance both growth- and performance-oriented demands. These results contribute to a broader understanding that growth mindset interventions are most effective when they are embedded in a supportive context (Yeager and Dweck 2020). Future research and policy initiatives should focus on integrating these contextual elements to optimize growth mindset interventions across diverse educational settings.

### 4.3. Limitations and Future Research

While this study contributes valuable insights into how supportive contexts moderate the impact of a student growth mindset on academic achievement, several limitations must be acknowledged. First, the cross-sectional design limits our ability to make causal inferences. The data were all captured at one point in time, and there is no evidence that the measures were assessed prior to final exam scores. This limitation prevents us from determining the causal direction of the relationships. For instance, it is possible that higher student grades could contribute to a higher growth mindset score, rather than the reverse. Most importantly, there is no experimental control, and thus all we have are correlations, leaving the possible causal direction ambiguous. While we identified significant moderation effects, it remains unclear how these relationships evolve over time. Future research should adopt longitudinal studies to track changes in students’ growth mindsets and academic performance across different educational stages, which would enable a better understanding of the long-term impact of these supportive contexts (Maxwell and Cole 2007). Second, this study primarily uses academic performance, as reflected by standardized test scores, to evaluate students’ outcomes. Future research could incorporate additional measures, such as self-efficacy, motivation, and learned helplessness, to provide a more comprehensive understanding of academic performance. Third, the sample used in this study, consisting of middle school students from Shanghai, may limit the generalizability of the findings to other cultural or educational contexts. Research has suggested that the effects of growth mindset interventions can vary significantly across different cultures and educational systems (Zhang and He 2024). Cross-cultural studies are needed to determine whether similar patterns of moderation hold in other regions, particularly in contexts with different levels of academic pressure or varying degrees of parental involvement. Fourth, while this study explored key moderating variables, other important factors, such as peer influence, socioeconomic status, and school resources, were not examined. Future studies should include these variables to obtain a more comprehensive understanding of how the growth mindset interacts with a variety of contextual factors. For example, peer influence could play a significant role in shaping student attitudes toward learning and challenges, whereas socioeconomic status might influence access to educational resources that promote the development of a growth mindset (Claro et al. 2016). Finally, this study exclusively employed quantitative methods, which, while useful for statistical analysis, may not fully capture the complex experiences of students and teachers in real-world settings. Qualitative methods such as interviews or classroom observations could provide deeper insights into how teacher mindset and parental support are expressed in everyday interactions with students (Creswell and Plano Clark 2017). Employing a mixed-method approach in future research could lead to richer, more nuanced findings that better reflect the lived experiences of students.

Building on the limitations outlined above, future research should also consider including additional variables to better capture the interplay of factors influencing growth mindset. Variables such as self-efficacy, intrinsic motivation, and emotional regulation could provide further insights into the psychological mechanisms underpinning growth mindset development and its impact on academic achievement. For instance, self-efficacy plays a critical role in shaping students’ beliefs about their abilities and their persistence through challenges (Wasylkiw et al. 2020). Moreover, intrinsic motivation may strengthen the link between growth mindset and academic outcomes by fostering greater engagement and perseverance in learning (Bedford 2017). Incorporating such variables could offer a more holistic understanding of the factors driving growth mindset, enriching theoretical frameworks and guiding the development of more targeted, context-sensitive interventions across diverse educational settings.

## 5. Conclusions

This study highlights the role of supportive contexts—teacher mindset, perceived school climate, and parental autonomy support—in moderating the relationship between students’ growth mindset and their academic achievement. The findings suggest that when students perceive supportive teaching environments, positive school climates, and autonomy-promoting parental behaviors, the associations between growth mindset and academic performance may be amplified. This underscores the importance of embedding growth mindset interventions within broader ecological systems to enhance their relevance and applicability. Future research should examine how these contextual factors interact over time and across diverse cultural settings to provide a deeper understanding of how to refine growth mindset interventions in various educational contexts. By fostering environments that support both academic growth and personal development, educators and parents may help sustain students’ motivation, resilience, and engagement with learning tasks.

## Figures and Tables

**Table 1:** Descriptive statistics, reliability estimates, and intercorrelations of the study variables (teachers’ growth-mindset beliefs).

Variables	Mean (*SD*)	1	2	3	4	5
1. Age	13.38 (2.20)	-				
2. Sex	-	0.04	-			
3. Education (in years)	7.80 (1.70)	0.05	0.04	-		
4. Students’ growth-mindset beliefs	5.21 (1.57)	−0.03	0.05	0.05	(0.83)	
5. Teachers’ growth-mindset beliefs	5.15 (1.32)	0.02	0.02	0.04	0.48 **	(0.82)
6. Academic achievement	82.18 (9.18)	0.03	0.05	0.02	0.58 ***	0.29 *

Notes. *n* = 358. The diagonal values in parentheses represent the alpha-reliability coefficients. * *p* < 0.05, ** *p* < 0.01, *** *p* < 0.001.

**Table 2:** Descriptive statistics, reliability estimates, and intercorrelations of the study variables (perceived school climate).

Variables	Mean (*SD*)	1	2	3	4	6	7	8	9	10
1. Age	13.38 (2.20)	-								
2. Sex	-	0.04	-							
3. Education (in years)	7.80 (1.70)	0.05	0.04	-						
4. Students’ growth-mindset beliefs	5.21 (1.57)	−0.03	0.05	0.05	(0.83)					
5. Teacher–student relations	2.87 (0.42)	0.04	0.03	0.05	0.37 **	(0.81)				
6. Student–student relations	3.19 (0.75)	0.05	0.04	0.04	0.19 *	0.15 *	(0.84)			
7. Fairness of rules	3.09 (0.52)	0.04	0.03	−0.02	0.23 *	0.29 **	0.06	(0.82)		
8. School safety	2.58 (0.81)	0.06	0.04	0.04	0.15 *	0.31 **	0.19 *	0.03	(0.86)	
9. Liking of school	2.92 (0.41)	0.03	0.06	0.05	0.35 **	0.42 **	0.22 *	0.25 *	0.21 *	
10. Academic achievement	82.18 (9.18)	0.03	0.05	0.02	0.58 ***	0.12 *	0.06	0.06	0.03	−0.05

Notes. *n* = 358. The diagonal values in parentheses represent the alpha-reliability coefficients. * *p* < 0.05, ** *p* < 0.01, *** *p* < 0.001.

**Table 3:** Descriptive statistics, reliability estimates, and intercorrelations of the study variables (parental autonomy support).

Variables	Mean (*SD*)	1	2	3	4	5	6	7	8	9	10
1. Age	13.38 (2.20)	-									
2. Sex	-	0.04	-								
3. Education (in years)	7.80 (1.70)	0.05	0.04	-							
4. Students’ growth-mindset beliefs	5.21 (1.57)	−0.03	0.05	0.05	(0.83)						
5. Choice within certain limits	4.66 (0.53)	0.03	0.05	0.04	0.29 *	(0.82)					
6. Rationale for demands and limits	4.31 (0.61)	0.04	−0.04	0.06	0.31 **	0.33 **	(0.85)				
7. Acknowledgment of feelings	4.78 (0.83)	0.05	0.05	0.03	0.26 *	0.23 *	0.36 **	(0.83)			
8. Threats of punishment	6.04 (1.13)	0.04	0.06	0.05	−0.41 **	−0.21 *	−0.24 *	−0.25 *	(0.83)		
9. Performance pressure	6.53 (1.21)	−0.05	0.04	0.05	−0.45 **	−0.39 **	−0.41 **	−0.40 **	0.39 **	(0.85)	
10. Guilt-inducing criticism	5.41 (0.93)	0.02	0.05	−0.03	−0.42 **	−0.26 *	−0.27 *	−0.31 **	0.27 *	0.23 *	(0.84)
11. Academic achievement	82.18 (9.18)	0.03	0.05	0.02	0.58 ***	0.06	0.05	0.05	−0.08	−0.18 *	−0.05

Notes. *n* = 358. The diagonal values in parentheses represent the alpha-reliability coefficients. * *p* < 0.05, ** *p* < 0.01, *** *p* < 0.001.

**Table 4:** Results of regression analyses for testing the moderating effect of teachers’ growth mindset.

	Academic Achievement		
	M1	M2	M3
Step 1			
Age	0.02	0.02	0.02
Sex	0.01	0.01	0.01
Educational years	0.03	0.03	0.03
Step 2			
Students’ growth mindset		0.29 ***	0.29 ***
Step 3			
Students’ growth mindset × teachers’ growth mindset			0.18 **
*R* ^2^	0.02	0.14 **	0.23 **
*F*	3.91	40.33 ***	51.64 ***
Δ*R*^2^	--	0.12 **	0.09 **
Δ*F*	--	27.37 **	12.51 **

Notes: *n* = 358. ** *p* < 0.01, *** *p* < 0.001.

**Table 5:** Results of regression analyses for testing the moderating effect of perceived school climate.

	Academic Achievement		
	M1	M2	M3
Step 1			
Age	0.02	0.02	0.02
Sex	0.01	0.01	0.01
Educational years	0.03	0.03	0.03
Step 2			
Students’ growth mindset		0.29 ***	0.29 ***
Step 3			
Students’ growth mindset × teacher–student relations			0.16 **
Students’ growth mindset × student–student relations			0.11 **
Students’ growth mindset × fairness of rules			0.13 **
Students’ growth mindset × school safety			0.11 **
Students’ growth mindset × liking of school			0.15 **
*R* ^2^	0.02	0.14 **	0.24 **
*F*	3.91	40.33 ***	53.21 ***
Δ*R*^2^	--	0.12 **	0.10 **
Δ*F*	--	27.37 **	16.05 **

Notes: *n* = 358. ** *p* < 0.01, *** *p* < 0.001.

**Table 6:** Results of regression analyses for testing the moderating effect of parental autonomy support.

	Academic Achievement		
	M1	M2	M3
Step 1			
Age	0.02	0.02	0.02
Sex	0.01	0.01	0.01
Educational years	0.03	0.03	0.03
Step 2			
Growth mindset		0.29 ***	0.29 ***
Step 3			
Students’ growth mindset × choice within certain limits			0.12 **
Students’ growth mindset × rationale for demands and limits			0.12 **
Students’ growth mindset × acknowledgment of feelings			0.16 **
Students’ growth mindset × threats of punishment			−0.11 **
Students’ growth mindset × performance pressure			−0.12 **
Students’ growth mindset × guilt-inducing criticism			−0.11 **
*R* ^2^	0.02	0.14 **	0.27 **
*F*	3.91	40.33 ***	58.71 ***
Δ*R*^2^	--	0.12 **	0.13 **
Δ*F*	--	27.37 **	32.61 **

Notes: *n* = 358. ** *p* < 0.01, *** *p* < 0.001.

## Data Availability

The data presented in this study are available on request from the corresponding author due to privacy and ethical restrictions.
